# The $^{146}\text{Sm}$ half-life re-measured: consolidating the chronometer for events in the early Solar System

**DOI:** 10.1038/s41598-024-64104-6

**Published:** 2024-08-01

**Authors:** Nadine M. Chiera, Peter Sprung, Yuri Amelin, Rugard Dressler, Dorothea Schumann, Zeynep Talip

**Affiliations:** 1 Center of Nuclear Engineering and Science, Paul Scherrer Institut, Forschungsstrasse 111, Villigen-PSI, 5232 Switzerland; 2 Research School of Earth Sciences, The Australian National University, 142 Mills Road, Acton, ACT 0200 Australia

**Keywords:** $^{146}$Sm half-life, Isotopic dating, Early solar system isotopic chronometer, MC-ICP-MS, TIMS, $\alpha$-Spectrometry, Early solar system, Inorganic chemistry, Nuclear chemistry, Geochemistry

## Abstract

The half-life of the extinct radiolanthanide $^{146}\text{Sm}$, important for both geochronological and astrophysical applications, was re-determined by a combination of mass spectrometry and $\alpha$-decay counting. Earlier studies provided only limited information on all potential factors that could influence the quantification of the half-life of $^{146}\text{Sm}$. Thus, special attention was given  here to a complete documentation of all experimental steps to provide information about any possible artifacts in the data analysis. The half-life of $^{146}\text{Sm}$ was derived to be 92.0 Ma ± 2.6 Ma, with an uncertainty coverage factor of $\mathsf{\textit{k}\!=\!1}$.

## Introduction

Nuclear decay data are the most prominent characteristic of radioactive isotopes. In recent decades, high-precision measurements of the nuclear decay properties of rare or hard-to-measure isotopes have been performed. The main focus of these experiments was to confirm or rectify existing data with significant smaller uncertainties. Recently, the re-measured half-lives of a considerable number of radionuclides—such as $$^{60}\text{Fe}$$^1–3^, $$^{79}\text{Se}$$^4,5^, $$^{93}\text{Mo}$$^6^, $$^{209}\text{Po}$$^7–9^—showed substantial disagreements with the previous values. These discrepancies have significant consequences, such as, in the determination of timescales of galactic events, in the interpretation of nucleosynthetic processes in the Milky Way or in the evaluation of nuclear waste repositories.

An exceptional example of the effects that a reassessment of the half-life can have is the case of  $^{146}\text{Sm}$. With its pure $\alpha$-decay, the $^{146}\text{Sm}$–$^{142}\text{Nd}$ system is used as an isotopic chronometer for dating^10–12^. Specifically, it is used for dating the geochemical processes responsible for the chemical differentiation of the Earth^13–16^, lunar volcanism^17–19^, and meteoritic samples^20–24^. Therefore, an accurate value for the half-life of $^{146}\text{Sm}$ is of crucial importance, as it determines the correctness and quality of the dating *via* the $^{146}\text{Sm}$–$^{142}\text{Nd}$ isotope chronometer. However, two different half-lives ($t_{1/2}$) for $^{146}\text{Sm}$ have been used by the scientific community over the last ten years: $t_{1/2}\!=\!{103(4)}\,\hbox {Ma}$ from averaging the values of^25,26^ and $t_{1/2}\!=\!{68(7)}\,\text{Ma}$, reported in^27^. An abbreviated representation of the uncertainty values is used here. The uncertainty is added in brackets in units of the last significant digit of the given value, e.g. 103(4) Ma displays a half-life of (103±4)$\cdot 10^6$ a. Consequently using the presumed shorter half-life of $^{146}$Sm leads to an $\approx {30}\%$ smaller time interval of early planetary differentiation events derived from the $^{146}$Sm–$^{142}$Nd isotope chronometer compared to using the longer half-life. Moreover, various conflicting results either from theoretical calculations (see e.g.^28–38^) or from data extrapolations^39^ further emphasize this pronounced discrepancy between the two experimental half-lives. The theoretical attempts have shown that the predictive power of the systematic or semi-empirical half-life formula is very limited and cannot be used to refute experimental data.

With the aim to clarify the situation, the IUPAC-IUGS Joint Task Group “Isotopes in Geosciences”^40^ recently re-evaluated all $^{146}\text{Sm}$ half-life measurements performed in the past. However, the incomplete documentation of systematic bias effects in most of the available studies made the review of the published data difficult, thus a well-justified recommendation for the most accurate half-life value was not possible. Therefore, it was recommended that publications using the radiometric pair $^{146}\text{Sm}$–$^{142}\text{Nd}$ should include a double set of calculations using both half-lives, 103(4) Ma and 68(7) Ma, e.g., in^41^.

After more than a decade, the scientific community has gained some clarity with the retraction^42^ of the shorter value published by Kinoshita et al.^27^, which was triggered by the discovery of inconsistencies in processing the samarium samples during the measurements. However, the elimination of one of the two half-life values does not *per se* prove that the remaining one is accurate. An independent new measurement of the half-life of $^{146}\text{Sm}$ is urgently needed.

To measure the half-life of such a radionuclide, many restrictions have to be overcome. The main difficulty is to obtain samples of the wanted isotope in sufficient quantity and purity. This major challenge is exacerbated by inherent problems in performing accurate and precise measurements. To overcome the first limitation, the long-term project “ERAWAST—Exotic Radionuclides from Accelerator Waste for Science and Technology”^43,44^ was launched at the Paul Scherrer Institut (PSI), with the aim of extracting exotic radionuclides for scientific applications from radioactive material produced on-site at the accelerator facilities.

In this initiative, special attention was given to improving the nuclear databases by redetermining the half-life of exotic radionuclides such as $$^{148}\text{Gd}$$^45^, $$^{154}\text{Dy}$$^46^, and $$^{146}\text{Sm}$$, the later reported here. Since the expected half-life of $$^{146}\text{Sm}$$ is of the order of 100 Ma, it is not possible to monitor the decay of the activity of a sample *in situ*. Therefore, the half-life must be quantified using the so-called “direct method”, which consists of determining the number of $$^{146}\text{Sm}$$ atoms *N* in a sample in combination with the measurement of the $$^{146}\text{Sm}$$ activity *A* according to the following equation:

1 $$\begin{aligned} t_{1/2} = ln(2) \times \left( \frac{N}{A}\right) \end{aligned}$$

## General procedure

Highly activated tantalum specimens with a total dose rate of more than 10 mSv/h from an irradiation program for materials science at PSI^47^, were used as the source of $^{146}\text{Sm}$. Using radiochemical separation and purification methods, namely a sequence of five consecutive ion chromatographic separations with four different ion exchange resins^48^, a samarium fraction containing ppb amounts of $^{146}\text{Sm}$ in nitric acid medium (hereafter referred to as the “Sm master-solution”) was obtained by processing several of these tantalum samples. The performance of the chromatographic separations was monitored with different radiolanthanides, which ensure an accurate localization of the elution fraction containing only samarium. To accurately monitor the samarium concentration during the separation procedures, and all subsequent treatments, $^{145}\text{Sm}$ was added to the samarium solution as an internal $\gamma$-ray emitting radiotracer. The applied method generally guarantees baseline separated chromatograms of the lanthanides, showing a suppression of the neighboring elements by a factor of 100 at least. In particular, it was shown in^48^ that the neodymium impurity in the samarium fraction, which contributes to the 146-mass isobar, could be suppressed by at least a factor of 1000 with respect to $^{146}\text{Sm}$.

After the Sm master-solution was prepared, gravimetrically quantified aliquots were taken for the determination of the $^{146}\text{Sm}$ concentration by multicollector inductively coupled plasma mass-spectrometry (MC-ICP-MS) without any further chemical treatment. The reverse isotope dilution mass-spectrometry^49^ (IDMS) and the internal standard method coupled with a gravimetric standard addition method (ISM-SAM)^50^ were applied. Additionally, a $^{146}\text{Sm}$ sample for $\alpha$-spectrometric measurement was prepared. For this purpose, another gravimetrically determined aliquot of the Sm master-solution was taken and used for the deposition of a thin and uniform layer using the molecular plating (MP) technique (also referred to as “electrodeposition”)^51^. The solution used for MP is referred to in the following as the “Sm plating-solution”. Graphite was chosen as the deposition substrate due to its general chemical inertness.

The deposition yield of samarium was determined by monitoring the activity of the added $^{145}\text{Sm}$ radiotracer in the aliquot of the Sm master-solution before deposition and subsequently in the deposited layer on the graphite foil. Both $\gamma$-spectrometric measurements were performed in matching geometric configurations using the same methodology and setup as described in^46^. After the MP procedure, the $^{146}\text{Sm}$ activity was quantified by $\alpha$-spectrometry. The $\alpha$-measurement was performed for 58 d at a defined solid angle. The absolute activity of the $\alpha$-source was deduced by directly comparing the count rate measured for $^{146}\text{Sm}$ against the count rate of an $^{241}\text{Am}$ standard reference source with a certified activity. To minimize any geometric differences between the $\alpha$-spectrometric measurements of $^{146}\text{Sm}$ and $^{241}\text{Am}$, the area of deposited activities of both $\alpha$-sources had the same diameter. In addition, custom-made sample holders were used for both $\alpha$-measurements to ensure an identical distance between the sample surface and the detector.

The isotopic composition and concentration of the Sm plating-solution after MP was determined using an independent and complementary method, the thermal ionization mass spectrometry (TIMS). The sample for TIMS (hereafter referred to as “Sm TIMS-solution”) was prepared by collecting the remaining Sm plating-solution and converting it to a nitric acid form. This additional mass spectrometry step acts as an independent confirmation of the number of $^{146}\text{Sm}$ atoms in the Sm master-solution. Furthermore, if the isotope ratios of samarium remain the same in both the Sm master-solution and the Sm TIMS-solution, it can be confirmed that no significant amount of samarium was added from external sources while the MP treatment. However, due to the chemical treatments performed during the preparation of the Sm plating-solution, the $^{146}\text{Sm}$ concentrations of the Sm master-solution (obtained by MC-ICP-MS) and the Sm TIMS-solution (measured by TIMS) will be different. Regardless, the results of the MC-ICP-MS and TIMS measurements can be interrelated by comparing the specific activity of $^{145}\text{Sm}$ in both solutions. For this purpose, a $\gamma$-spectrometric measurement also of the Sm TIMS-solution was performed.

**Figure 1:**
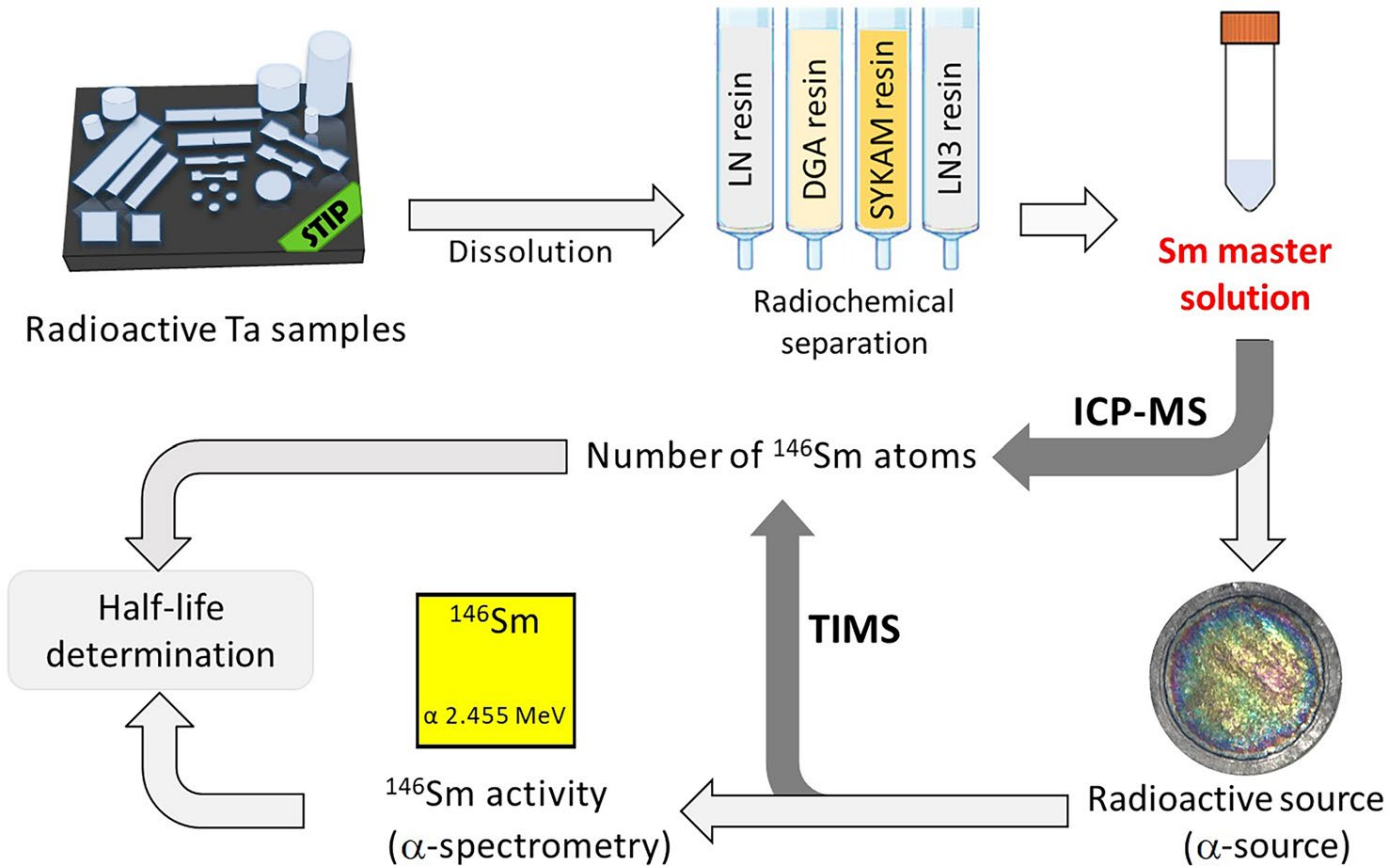
Schematic flowchart of the experimental steps followed in this work for the determination of the half-life of $^{146}\text{Sm}$ via the “direct method”.

The experimental steps for the re-determination of the half-life of $^{146}\text{Sm}$ are shown schematically in Fig. 1. Details of each technique used can be found in the section “Materials and methods”, with further experimental details provided in the Appendix with Supplementary information (“SI Appendix”).

Unless otherwise noted, all uncertainties reported here were calculated according to the recommendations of the Guide to the expression of Uncertainty in Measurement (GUM)^52^ and are given as combined standard uncertainties with a coverage factor $k\!=\!1$.

## Results

The aim of the $\alpha$-spectrometric measurements was to accurately determine the specific $^{146}\text{Sm}$ activity in the Sm master-solution, whereas the $\gamma$-spectrometric measurements of the radiotracer $^{145}\text{Sm}$ ($t_{1/2}\!=\!{340(3)}\,\text{d}$, $E_{\gamma}\!=\!{61.2}\,\text{keV}$^53^) were performed to ensure the quantitative traceability of all aliquots prepared from the Sm master-solution. The $\gamma$-measurement of the aliquot of the Sm master-solution ($\gamma$-measurement_A) before MP, which was later used as Sm plating-solution, showed a count rate of 8.526(50) cps for $^{145}\text{Sm}$ on June 24, 2021. The selected experimental parameters, namely the organic solvent, applied voltage, current, and duration of MP, were intentionally chosen to minimize the thickness of the $^{146}\text{Sm}$ layer to avoid irregular film formation or detachment of the samarium layer from the deposition substrate due to hydrogen production. At the same time, a suitable count rate for the activity measurements and an excellent $\alpha$-spectroscopic resolution must be guaranteed. Systematic pre-experiments have shown that a significant increase in deposition yield can only be achieved at the expense of the quality of the $\alpha$-sample. For this reason, a low deposition yield was accepted to ensure the best possible measurement result. The deposition layer obtained for $\alpha$-spectrometry clearly show second-order interference colors (see Fig. 1) indicating a layer thickness below 500 nm. After the MP procedure, the $\gamma$-activity of the $^{145}\text{Sm}$ radiotracer deposited on the graphite foil ($\gamma$-measurement_B) was determined on August 11, 2021 with 0.12462(54) cps. Taking into account the radioactive decay of the radiotracer $^{145}\text{Sm}$ between the two $\gamma$-spectrometric measurements (i.e., 48 d), the samarium deposition yield of 1.684(27)% was derived. The latter includes an additional correction factor (for details see^46^), which allows a more accurate comparison between the activity of $^{145}\text{Sm}$ contained in a solid deposited layer and in a liquid medium. The uncertainty in the half-life of $^{145}\text{Sm}$ was taken into account and included in the total uncertainty budget.

**Figure 2:**
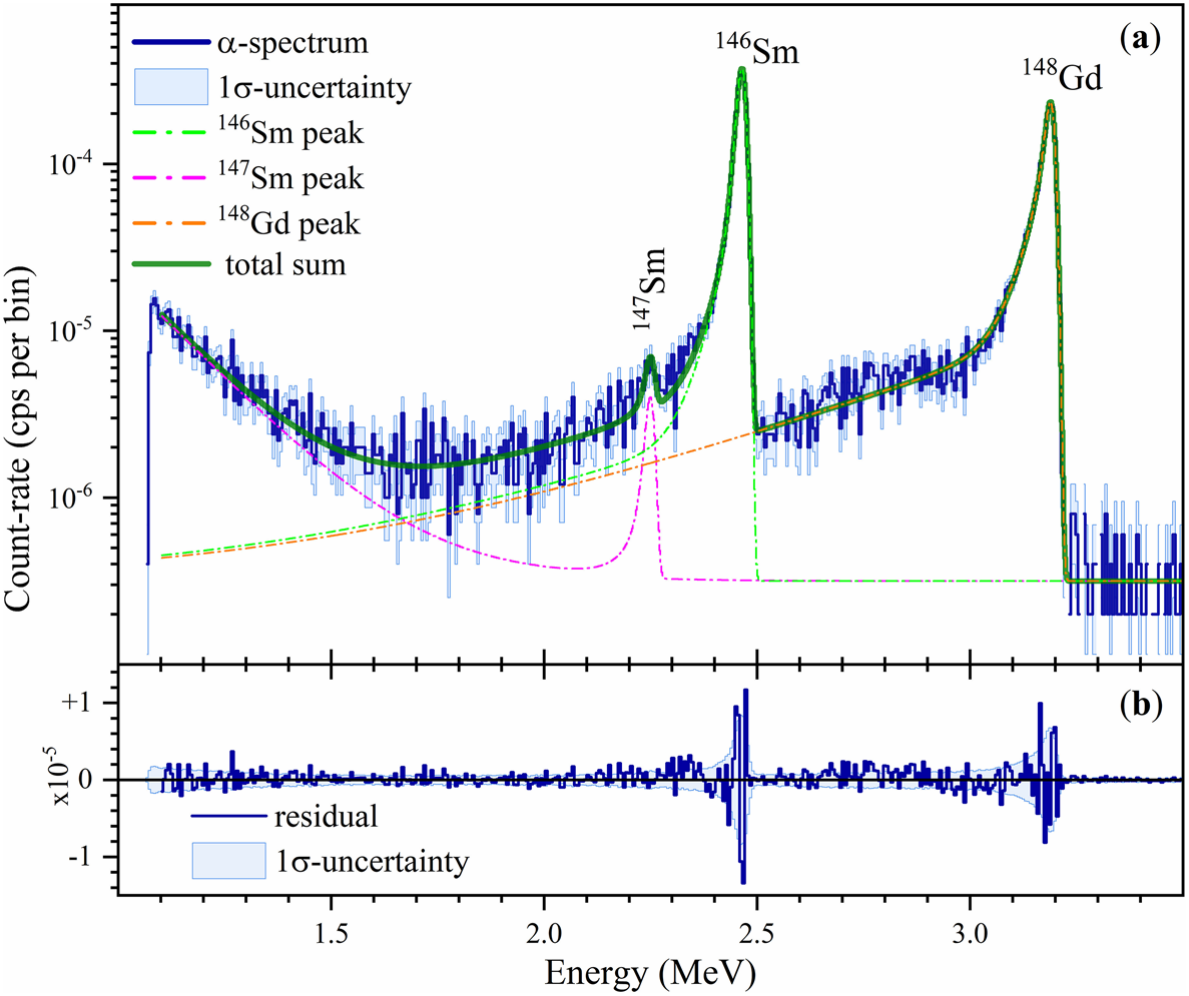
(**a**) Measured $\alpha$-spectrum (dark blue histogram) of the plated samarium on a graphite foil together with its 1$\sigma$-confidence interval (light blue shaded area). The total counting time was 5$\cdot 10^{6}$ s or 58 d. The histogram bin size is 5.713 keV. The peak-fitting model includes the $^{146}\text{Sm}$ peak (green dashed dotted line), the $^{148}\text{Gd}$ peak (red dashed dotted line) and the $^{147}\text{Sm}$ peak (purple dashed dotted line) which is superimposed with a low-energy electronic noise and a background component. The sum of all these components is shown as a dark green line. (**b**) Residuals of the peak fit (dark blue histogram) are consistent with the Poisson count statistics of the spectrum (1$\sigma$-uncertainty, light blue shaded area).

By applying a peak deconvolution method^54^ to fit the peaks (see SI Appendix Sect. SI 3) in the energy range 1.1 MeV to 3.5 MeV, the activity *A* of the molecular plated $^{146}\text{Sm}$ ($E_{\alpha}\!=\!{2.455(4)}\,\text{MeV}$^55^) was determined as 15.00(31) mBq (see Fig. 2 and SI Appendix Tables SI 10, SI 11, and SI 12). In addition, $^{147}\text{Sm}$ ($E_{\alpha}\!=\!{2.235(3)}\,\hbox{MeV}$^55^) and $^{148}\text{Gd}$ ($E_{\alpha}\!=\!{3.182680(24)}\,\text{MeV}$^55^), were identified (see Fig. 2), which are simultaneously produced by spallation of tantalum. The FWHM of the $^{146}\text{Sm}$ and $^{148}\text{Gd}$ $\alpha$-peak was found to be 26.9 keV and 28.3 keV, respectively.

Since isotopes of the same element behave chemically identical, it is not possible to remove $^{147}\text{Sm}$ from the Sm master-solution by chemical means. However, the contribution of the $^{147}\text{Sm}$ $\alpha$-decay to the $^{146}\text{Sm}$ $\alpha$-peak was found to be only 0.148(32) mBq or 0.99(21)%. Although $^{148}\text{Gd}$ was largely chemically separated, trace amounts were still detectable in the $\alpha$-spectrum due to its relatively short half-life of only 86.9(39) a^45^. From the $\alpha$-spectrum, an activity of 15.97(34) mBq was derived for $^{148}\text{Gd}$ in the plated sample, which corresponds to a concentration of $\approx\!{10^{-15}}\,\text{mol/g}$ $^{148}\text{Gd}$ (or 6$\cdot 10^8$ at/g) in the Sm master-solution. This value is significantly below the detection limit of all mass spectrometric methods used in this work. The specific $^{146}\text{Sm}$ activity 0.1416(37) Bq/g of the Sm master-solution was determined taking into account the used 6.293167(20) g of this solution and the derived samarium deposition yield of 1.684(27)%.

The concentration of $^{146}\text{Sm}$ in the Sm master-solution was determined using both MC-ICP-MS and TIMS. For MC-ICP-MS, the isotope ratio $^{147}\text{Sm}$/$^{146}\text{Sm}$ of 9.061(11) was deduced from measurements where no samarium reference standards were added. The concentration of $^{146}\text{Sm}$ in the Sm master-solution was determined to be 0.9748(98) nmol/g using ISM-SAM and 0.9900(54) nmol/g using the IDMS technique. The averaged value of 0.982(15) nmol/g was used as the MC-ICP-MS result for further calculations (Table 1).

**Table 1:** Results of the $\alpha$-activity and mass-spectrometric measurements to determine the specific activity *A* (given in Bq/g), the $^{146}\text{Sm}$ concentration (given in nmol/g), and the number of atoms *N* per g of Sm master-solution.

Parameter	Method	Result
*A*	$\alpha$-spectrometry	0.1416(37) Bq/g
	MC-ICP-MS standard addition	0.9748(98) nmol/g
	MC-ICP-MS isotope dilution	0.9900(54) nmol/g
	MC-ICP-MS average	0.9824(150) nmol/g
	TIMS	0.9848(73) nmol/g
	MC-ICP-MS and TIMS average	0.9836(87) nmol/g
*N*	Number of $^{146}\text{Sm}$ atoms	5.924(52)$\cdot 10^{14}$ at/g

The concentration of $^{146}\text{Sm}$ in the Sm master-solution was also measured using TIMS. The isotope ratio $^{147}\text{Sm}$/$^{146}\text{Sm}$ was determined to be 9.0618(93) by using five loads with different amounts of the Sm TIMS-solution, and the $^{146}\text{Sm}$ concentration was quantified using the IDMS technique to be 0.36378(36) nmol/g. As mentioned above, this value differs significantly from the concentration of $^{146}\text{Sm}$ in the Sm master-solution due to the chemical treatments during MP. However, the perfect agreement of the $^{147}\text{Sm}$/$^{146}\text{Sm}$ isotope ratio obtained by TIMS and MC-ICP-MS proves that no significant amounts of natural samarium were introduced from external sources during MP and the preparation of the Sm TIMS-solution. Therefore, it was possible to establish an interrelation between the $^{146}\text{Sm}$ concentration in the Sm TIMS-solution and in the Sm master-solution by comparing their $^{145}\text{Sm}$ $\gamma$-activities evaluated at the same reference date and account for the concentration change in the Sm TIMS-solution. From the results of $\gamma$-measurement_C, a specific $^{145}\text{Sm}$ count rate in the Sm TIMS-solution of 0.29936(31) cps/g was determined on March 3, 2022. The specific count rate of $^{145}\text{Sm}$ in the Sm master-solution was calculated for this date from $\gamma$-measurement_A to be 0.8104(59) cps/g. Considering the TIMS result, the concentration of $^{146}\text{Sm}$ in the Sm master-solution is 0.9848(73) nmol/g, which is in excellent agreement with the averaged result of the MC-ICP-MS analyses. The concentration of $^{146}\text{Sm}$ in the Sm master-solution (see Table 1) is determined as the mean value of the MC-ICP-MS and TIMS results, i.e. 0.9836(87) nmol/g.

By applying the obtained values for the number of atoms *N* and the activity *A* to Equation 1, a half-life $t_{1/2}\!=\!{92.0(26)}\,\text{Ma}$ of $^{146}\text{Sm}$ results with a combined standard uncertainty of 2.76%. The uncertainty budget for all used data is given in Table 2.

**Figure 3:**
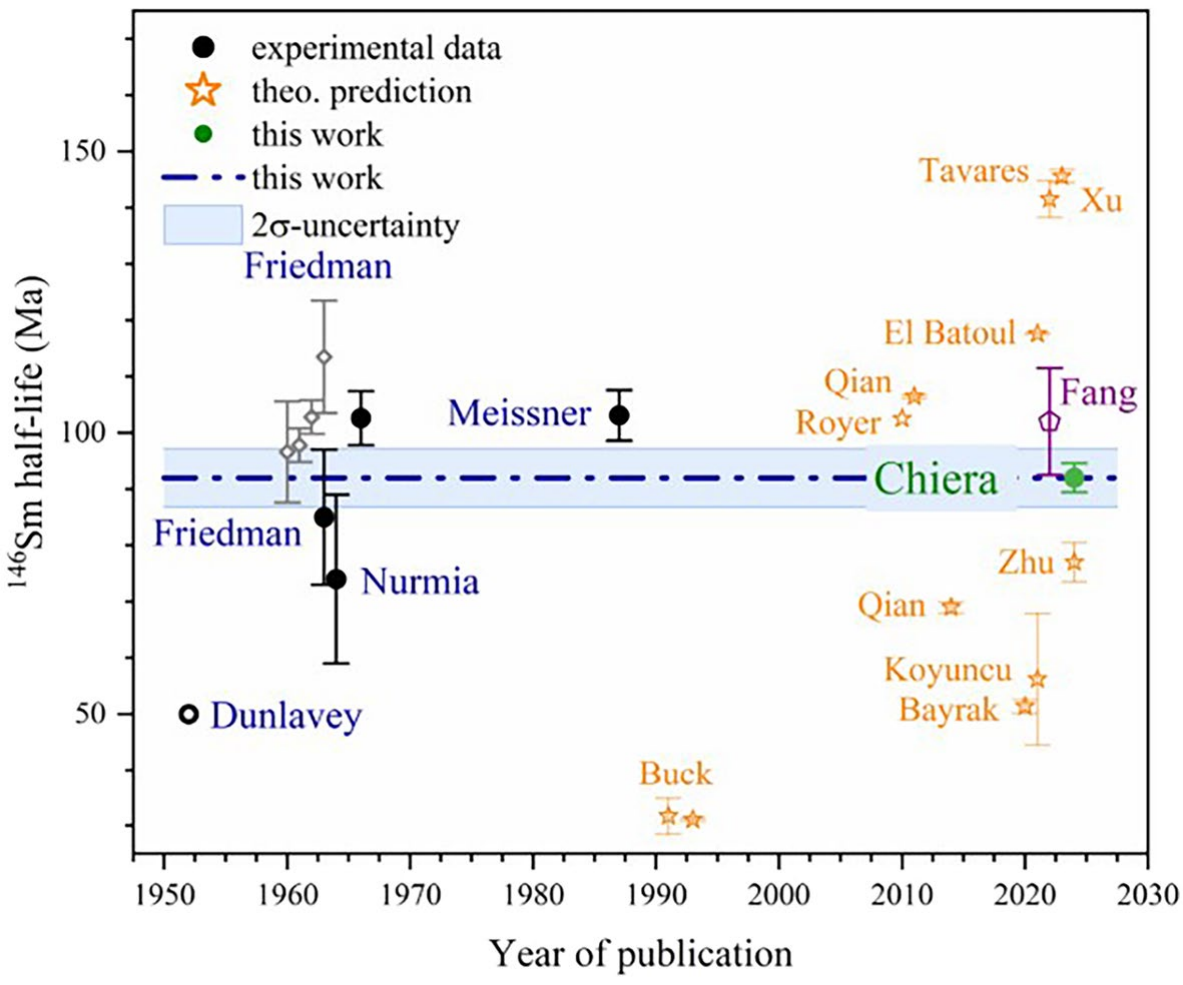
Experimental data (circles) and theoretical predictions (stars) for the $^{146}\text{Sm}$ half-life throughout the past decades. The half-live value measured here (green circle) is plotted in comparison to previous half-live determinations^25,26, 58–60^ (circles with the last name of the first author). In addition are the value by Fang et al.^39^ (open violet pentagon) from dating evaluation of different samples, some theoretical predictions^28–38^ (open orange stars), and the individual results of Friedman et al.^25^ presented as open gray diamonds in addition to the average value given in the paper. The value given by Dunlavey and Seaborg^58^ was reported without any uncertainty and is, therefore, displayed as open circle.

In Fig. 3, the result obtained here is shown alongside all other experimental half-life determinations (with the exception of the retracted value from Kinoshita et al.^27^) and some selected theoretical half-life predictions of $^{146}\text{Sm}$ in relation of the date of the respective publication. A detailed assessment of all previous measurements discussing possible short comings is given in^40^. The theoretical publications^28–38^ are restricted to half-life predictions not exceeding 150 Ma. Also, the attempt by Fang et al.^39^ to combine the age determination of various samples using different isotope chronometers and thereby perform a best-fit of the $^{146}\text{Sm}$ half-life could not sufficiently constrain this value to be suitable for a dating application.

For a reliable application of the $^{146}\text{Sm}$–$^{142}\text{Nd}$ isotope chronometer, an accurate determination of the $^{146}\text{Sm}$ half-life is an essential prerequisite. With the recent retraction of the Kinoshita value^42^ and the value determined here, the main problem has been addressed, leading to a significant improvement in the consistency of the data set. All previous half-life determinations were lacking in-depth documentation of the individual experimental steps, so that it is not possible to adequately consider possible systematic biases, as explained in detail in^40^. The present study contains an as complete as possible documentation of all experimental steps performed (see SI Appendix) and thus enables the assessment of possible artifacts. Based on this information, the half-life of $^{146}\text{Sm}$ was determined to be 92.0 Ma with an uncertainty of less than 3%.

Additional independent determinations of the $^{146}\text{Sm}$ half-life using alternative methods, such as the ongoing studies with metallic magnetic calorimeters or transition edge sensors^56,57^, will complement these findings and help to establish a precise and accurate half-life value for this important chronometer.

**Table 2:** Uncertainty budget for the determination of the half-life of  $^{146}\text{Sm}$. Combined standard uncertainties with a coverage factor $k\!=\!1$ are given. The partial uncertainty contributions of the half-lives of $^{133}\text{Ba}$ and $^{241}\text{Am}$ with 203 ppm and 10.5 ppm, respectively, as well as the contributions resulting from the weighing of 3.2 ppm are not shown in the table. These uncertainties have a total partial contribution of less than 0.0054%.

Parameter	Combined uncertainty	Source of uncertainty	Partial contribution
$^{146}\text{Sm}$ concentration in Sm master-solution	0.90%	MC-ICP-MS	7.6%
		TIMS	1.4%
		Scatter between MS results	1.5%
Samarium deposition yield	1.66%	Yield without correction	6.9%
		Correction factor	23.2%
		$^{133}\text{Ba}$ standard	3.8%
		$^{145}\text{Sm}$ half-life	2.0%
$^{146}\text{Sm}$ activity	2.03%	$^{146}\text{Sm}$ counting	33.6%
		$^{241}\text{Am}$ counting	6.5%
		$^{241}\text{Am}$ standard	13.5%
Total	2.76%		100.0%

## Materials and methods

In total, an amount of 6.732910(20) g of Sm master-solution was obtained from the chemical treatment (for more details see description of “Method B” in^48^) of the activated tantalum specimens (see SI Appendix Table SI 1).

### Molecular plating

In preparation for the MP procedure, the custom-made plating cell consisting of polytetrafluoroethylene (described in^46,61^) was cleaned by stepwise rinsing with 1 M HNO$_{3}$, 18.2 M$\Omega$cm milliQ-water and isopropanol. A platinum wire spiral was used as anode and likewise cleaned. The cathode, a block of copper, was cleaned with 0.1 M citric acid, washed with MilliQ water and rinsed with isopropanol. The graphite deposition foil (thickness: 75 $\mu$m, diameter of the deposition area: 20 mm, purity: 99.8%, Flexible Graphite, GoodFellow Cambridge Ltd.) was rinsed in isopropanol before MP.

For the MP procedure, a gravimetrically determined aliquot of 6.293167(20) g of the Sm master-solution (see SI Appendix Table SI 7) was filled into a custom-made polyether ether ketone vial (PEEK-vial). Before the MP procedure, the activity of the aliquot contained in the PEEK-vial was determined by a $\gamma$-spectrometric measurement ($\gamma$-measurement_A).

After the $\gamma$-measurement, the samarium solution contained in the PEEK-vial was reduced to dryness at 70$^\circ$C under a N$_2$ gas flow to remove any residual HNO$_3$ and water. This was a necessary step to prevent the development of H$_2$ at the cathode, which generally affects the quality of the deposited layer^62^. Subsequently, the obtained residue was dissolved in 15 mL methanol. Due to the polar and protic nature of methanol and its ability to dissolve lanthanide nitrates similar like water, this organic compound was chosen as both the dissolution and plating medium. The Sm plating-solution was added into the plating cell, where a thin samarium layer with a diameter of 20 mm was molecular plated onto the graphite foil by applying a constant voltage of 30 V for 30 min. During the plating process, the distance between the two electrodes was approximately 10 mm. To avoid inhomogeneities in the deposition layer due to temperature fluctuations during deposition, the cathode was equipped with a Peltier cooler that kept the graphite foil at a constant temperature of 15$^\circ$C.

### $\gamma$-Spectrometric measurements

All $\gamma$-spectrometric measurements of the $^{145}\text{Sm}$ radiotracer were performed with a Broad Energy Germanium (BEGe) $\gamma$-detector (Mirion Tech.—Canberra; crystal dimensions diameter: 61 mm, thickness: 25 mm). Data acquisition and analysis was performed using Genie 2000 Gamma Acquisition  & Analysis software (Mirion Tech.—Canberra). Energy calibration was done using a certified point-like $^{152}\text{Eu}$ source (Physikalisch-Technische Bundesanstalt - “PTB”). The full width at half maximum (FWHM) energy resolution was 0.57 keV at 61.25 keV. A dedicated sample holder was used to ensure the $\gamma$-spectrometric measurements in two geometrically equivalent positions as described in^46^ (see SI Appendix Fig. SI 1).

#### $\gamma$-Measurement_A

The aliquot of the Sm master-solution taken for MP was filled into the custom-made PEEK-vial (inner diameter: 20 mm, thickness at the bottom: 1.0 mm) and evaporated to dryness at 70$^\circ$C under N$_2$ gas flow. The residue was dissolved with 400 $\mu$L of 1 M HNO$_3$. The PEEK-vial was placed on the lower stage of the sample holder in the Configuration_A (see SI Appendix Fig. SI 1). Additionally, a graphite deposition foil identical to the one used for MP was inserted between the bottom of the PEEK-vial and the detector endcap. The $\gamma$-spectrometric measurement of the $^{145}$Sm contained in the PEEK-vial was performed for 3585 s live-time, i.e., the dead time corrected counting period (see SI Appendix Fig. SI 2 and, Table SI 8). The samarium solution in the PEEK-vial was then used for MP.

#### $\gamma$-Measurement_B

The $^{145}\text{Sm}$ activity contained in the deposition layer after MP was measured by placing the foil in Configuration_B of the custom-made sample holder (see SI Appendix Fig. SI 1). A PEEK-disk (thickness: 1.0 mm, identical to the bottom of the PEEK-vial) was placed on the bottom stage of the sample holder between the graphite foil and the detector endcap. The $\gamma$-spectrometric measurement was performed for 601015 s live-time (see SI Appendix Fig. SI 2 and, Table SI 8).

#### $\gamma$-Measurement_C

After finalizing the TIMS analysis, the remainder of the Sm TIMS-solution was transferred from the Teflon-FEP transport bottle into a PEEK-vial, identical as the one used for $\gamma$-measurement_A. The transferred mass was determined gravimetrically (see SI Appendix Table SI 6). The sample was dried at 70$^\circ$C under a N$_2$ gas flow and dissolved in 400 $\mu$L of 1 M HNO$_3$. The PEEK-vial was then placed in the upper stage of the sample holder in Configuration_A (see SI Appendix Fig. SI 1 and Fig. SI 3 Table SI 9). A graphite deposition foil, identical to that used for MP was inserted between the bottom of the PEEK-vial and the detector end-cap. The $\gamma$-spectrometric measurement of the $^{145}\text{Sm}$ contained in the PEEK-vial was carried out for 431254 s live-time (see SI Appendix Fig. SI 3 and, Table SI 9).

### $\alpha$-Spectrometric measurements

The Alpha-Analyst (Mirion Tech.—Canberra), an integrated $\alpha$-spectrometer equipped with a passivated implanted planar silicon (PIPS) detector (Model A-450-21AM Mirion Tech.—Canberra; sensitive surface: 450 mm$^2$; energy resolution FWHM: 21 keV), was used for the activity quantification of the pure $\alpha$-emitter $^{146}\text{Sm}$. The data acquisition was performed with the Genie 2000 Alpha-Analysis software (Mirion Tech.—Canberra). The energy calibration of the detector was performed with different $\alpha$-sources: a point-like mixed source of $^{148}\text{Gd}$ and $^{244}\text{Cm}$ and a certified point-like three-line source with $^{239}\text{Pu}$, $^{241}\text{Am}$, and $^{244}\text{Cm}$ (Amersham International plc). The efficiency calibration of the detector was performed with a certified $^{241}\text{Am}$ reference standard (PTB, calibration reference no. PTB-6.11-2016-1769, $A\!=\!{539(11)}\,\hbox{Bq}$ as of 01.11.2016 00:00:00 CET, $k\!=\!2$), which had the same 20 mm diameter as the samarium deposition on the graphite foil ( see SI Appendix Fig. SI 4). Geometrical differences between the $^{241}\text{Am}$ standard source and the deposited samarium sample were further minimized by custom-made sample holders to ensure that the measurements were performed at the same distance of 10.4 mm between the sample surface and the passivated entrance window of the PIPS detector. The shape of the individual $\alpha$-peaks was parameterized according to a suggestion by Pommé and Marroyo^63^ (see SI Appendix Tables SI 10, 11, and 12).

### Mass-spectrometric measurements

#### MC-ICP-MS

All measurements were performed with the Nu Instruments Plasma 3 MC-ICP-MS at PSI. For the masses of 143, 145 to 155, 157, 158, and 161, the ion beams were collected simultaneously in Faraday cups connected to amplifier systems with a ${10^{11}}\,\Omega$ resistor in their feedback loop. $^{146}\text{Sm}$, which formed the weakest samarium ion beam, delivered a current between 0.3 pA and 0.6 pA over the entire three days of analysis. All analytes were introduced as solutions in 0.28 M HNO$_3$ using an Elemental Scientific APEX HF nebulizing system and a self-aspirating Elemental Scientific PFA-ST Microflow nebulizer consuming ca. 50 $\mu$L/min. The operating power of the plasma was 1350 W. The analytes were measured 9 times at a low mass resolution. Each analysis consisted of 60 signal integrations, each 7.5 s long.

Mixtures with different mass fractions of a reference samarium standard solution VHG-PSMN-100 (1000 $\mu$g/mL natural samarium in 5% HNO$_3$, LOT: 1018557-8 from LGC Limited with a certified content of 0.9990(20) mg/mL; hereinafter referred to as “Sm LGC-standard”) and a gadolinium reference standard solution Specpure (1000 mg/mL natural gadolinium in 5% HNO$_3$, LOT: 223965 from Thermo Scientific Chemicals with a certified content of 0.9900(150) mg/mL; hereinafter referred to as “Gd Specpure-standard”) with or without the addition of aliquots of the Sm master-solution was prepared and analyzed (see SI Appendix Tables SI 2, SI 3, and SI 4). The Gd-Sm solutions without Sm master-solution were used to characterize the relation between the extent of mass fractionation of gadolinium (interference-free $^{157}\text{Gd}$/$^{155}\text{Gd}$) and samarium (interference-free $^{147}\text{Sm}$/$^{149}\text{Sm}$).

The mass fractionation was corrected applying the exponential mass fractionation law^64^ by adding Gd Specpure-standard to the $^{146}\text{Sm}$ containing solutions. Mixed solutions of Sm LGC-standard and Gd Specpure-standard without aliquots of the Sm master-solution that were analyzed alongside the $^{146}\text{Sm}$ containing analytes served to characterized the relation between the magnitude of mass fractionation of gadolinium (interference-free $^{157}\text{Gd}$/$^{155}\text{Gd}$) and samarium (interference-free $^{147}\text{Sm}$/$^{149}\text{Sm}$). Assuming natural isotopy, the isobaric-interference-free gadolinium and samarium isotope ratios $^{157}\text{Gd}$/$^{155}\text{Gd}$ = 1.0566(12)^65^ and $^{147}\text{Sm}$/$^{149}\text{Sm}$ = 0.9213(12)^66^ were used to characterize the mass fractionation magnitude. Thus, all signals of $^{146}\text{Sm}$ containing analytes were corrected for mass fractionation. From this procedure, an isotope ratio $^{147}\text{Sm}$/$^{146}\text{Sm}$ of 9.061(11) was obtained.

*A priori*, there is no information about the isotopic abundances of neodymium in the $^{146}\text{Sm}$ containing analytes. A natural isotope abundance cannot be presumed due to the artificial production of neodymium isotopes by the activation of tantalum specimens. Thus, interference corrections could only be performed if the isotopy of neodymium is close to natural samples. Monitoring the signals at masses 143 and 145, which represent isobaric-interference-free neodymium isotopes, provides the possibility to evaluate the isotope ratios $^{143}\text{Nd}$/$^{146}\text{Sm}$ and $^{145}\text{Nd}$/$^{146}\text{Sm}$ (see SI Appendix Table SI 13) and thus assess the isotope ratio $^{143}\text{Nd}$/$^{145}\text{Nd}$. Note that this evaluation was only possible for the two analytes with the highest $^{146}\text{Sm}$ concentration, as the signals of $^{143}\text{Nd}$ and $^{145}\text{Nd}$ were generally close to and often below the quantification limit. This is a consequence of the chemical separation process, which guarantees a quantitative separation of neodymium and leads to a residual concentration that is at least 100 times lower than that of $^{146}\text{Sm}$. This has also been confirmed by independent measurements (see^48^). Consequently, only individual measurement runs in which the signals of both neodymium isotopes significantly exceeded the background values of 2 $\mu$V were used, resulting in an isotope ratio $^{143}\text{Nd}$/$^{145}\text{Nd}$ of 1.36(98). This value is consistent with the isotope ratio $^{143}\text{Nd}$/$^{145}\text{Nd}$ of natural sample 1.4682(14) (calculated from^67^). The interference corrections was therefore performed based on the $^{145}\text{Nd}$ signals assuming a natural isotopy of neodymium. Given the very low $^{145}\text{Nd}$ ion beam current in the sub-fA ranges, the calculated average contribution of neodymium to masses 146, 148, and 150 are 0.04% to 0.14%, 0.01% to 0.05%, and 0.01% to 0.05%, respectively. Thus, the inherent inaccuracies are estimated to be $\approx\!{0.035}\%$ at worst. Assuming natural neodymium abundances in the $^{146}\text{Sm}$ analytes to correct for interference is considered more accurate than not correcting for neodymium interference. The contribution of interfering $^{158}\text{Dy}$ to the overall signal at mass 158 was, at most $\approx\!{0.0002}\%$ and thus negligible. For this reason, no correction was made for isobaric interference due to $^{158}\text{Dy}$.

All reported uncertainties for the $^{146}\text{Sm}$ concentrations include the 0.2% uncertainty of the Sm LGC-standard. The uncertainty of both the IDMS method and the standard addition method is based on a Monte Carlo uncertainty propagation procedure that takes into account both weighing uncertainties and all measurement uncertainties of the respective inter- and intra-element isotope ratios. The reported average concentrations per method and their respective uncertainties cover the full range of possible results using all measurement data reported in SI Appendix Table SI 13. The uncertainty of the average of both methods includes the scatter 0.12% and the uncertainty propagation based on the uncertainties of the individual average values, about 1.0% and 0.55%, respectively (see SI Appendix Sect. SI 5). For all gravimetric steps, a yearly certified Mettler-Toledo XP56 balance (1 $\mu$g scale interval) was used in a temperature-controlled room at 20$^\circ$C to 23$^\circ$C.

#### TIMS

After the MP procedure, the remainder of the Sm plating-solution was collected and the methanol evaporated at room temperature under N$_2$ gas flow. The remaining residue was treated with a mixture of 11 M HNO$_3$ and 30% H_2_O_2_ (w/w in H$_{2}$O) to ensure the decomposition of any traces of organic solvent. The liquid was then evaporated at 70$^\circ$C under N$_2$ gas flow and the remaining dissolved in 1 M HNO$_3$.

The resulting Sm TIMS-solution was analyzed by TIMS without further purification. An aliquot of 0.51824(10) g was diluted with 0.5 M HNO$_3$ to a total weight of 102.84038(9) g in a 125 mL Teflon-FEP bottle. The isotopic composition and concentration of the Sm TIMS-solution were determined by TIMS on a modified MAT 261 at the Research School of Earth Sciences, The Australian National University.

The aim of these measurements was to determine the concentration of $^{146}\text{Sm}$ as precisely and accurately as possible. Thus, an established protocol for precise samarium and neodymium analysis from double filament loads (e.g.,^68^) was used. Sample solutions were evaporated with 20 $\mu$L of 0.007 M H$_3$PO$_4$, loaded in 1.0 $\mu$L of 0.5 M HNO$_3$ on outgassed rhenium filaments of a double filament (Re+Re) assembly, and analyzed using an established protocol for precise samarium and neodymium analysis from double filament loads (e.g.,^68^). The current of the ionization filament was set at 4.0 A and the current of the evaporation filament was varied between 0.8 A and 2.4 A. This allowed for the evaporation filament to achieve a stable ion beam at the maximum possible intensity lasting at least 2 h to 3 h. Since the Sm TIMS-solution has no known isotopic ratios that can be used for internal bias correction, the instrumental bias was corrected using the total evaporation and the incipient emission approaches that were discussed in detail by Amelin and Merle^69^. Two types of isotopic analyses after the TIMS measurements were performed, namely total evaporation (TE-TIMS) and incipient emission (IE-TIMS). For the measurements, a 6 Faraday cup configuration was used, where the samarium isotopes 146, 147, 149, and 152 were acquired, and possible neodymium interference was monitored on the masses 143 and 145.

Mainly the following measurements were performed. The original Sm TIMS-solution was used for a few measurements (5 loads) to determine the isotope ratio $^{147}\text{Sm}$/$^{146}\text{Sm}$. A natural samarium solution prepared from high purity samarium metal (purity of metal 99.996% from Ames Laboratory; hereafter referred to as “Sm Ames-solution”) was used as primary concentration standard and as supporting isotopic standard (6 loads). In addition, the synthetic samarium isotope mixture GBW04605 (from the series of eleven Certified Reference Materials “Samarium Isotopic Reference Material in Nitric Acid Solution, GBW04601–GBW04611” of the National Institute of Metrology of P.R. China of the National Institute of Metrology of P.R. China; hereafter referred to as “Sm GBW-solution”) was used as the primary isotope standard (5 loads). Mixtures of Sm TIMS-solution and Sm Ames-solution (6 analyzed mixtures, each 8 loads) were used for IDMS analysis (see SI Appendix Table SI 5). The size of the individual filament loads was between 1 ng and 100 ng samarium. The comparison between the bias correction with TE-TIMS and IE-TIMS showed a clear advantage of the TE-TIMS approach, which is consistent with the results of^69^ for the analysis of potassium by surface ionization with double filaments. The mass bias correction for the TIMS measurements was performed using the power-law approach (see^70^ and^71^). The TE-TIMS analyses of the Sm-Ames solution and the Sm-GBW solution using the isotope ratios $^{149}\text{Sm}$/$^{147}\text{Sm}$ and $^{152}\text{Sm}$/$^{147}\text{Sm}$ gave a bias coefficient of 0. 99932(39) (0.074%), while IE-TIMS from the same runs showed a value of 1.0072(73) (0.28%). The IE-TIMS values scatter more, leading to a higher uncertainty of the bias coefficient (see SI Appendix Fig. SI 5). Therefore, all mass bias corrections in this study, including the analyses of the IDMS mixtures, were carried out using the value obtained by TE-TIMS (see SI Appendix Table SI 14). This leads to an isotope ratio $^{147}\text{Sm}$/$^{146}\text{Sm}$ of 9.0618(93), in perfect agreement with the values $^{147}\text{Sm}$/$^{146}\text{Sm}$ of 9.061(11) meaured using MC-ICP-MS.

The calculated total uncertainty on the $^{146}\text{Sm}$ concentration includes: (1) the concentration uncertainty of Sm Ames-solution (0.01%); (2) the difference between the average concentrations calculated using the Sm master-solution and the dilute Sm TIMS-solution (0.077%); (3) the reproducibility of concentration calculations from a dilution series of five mixtures using Sm TIMS-solution (0.17%); and (4) the reproducibility of TE-TIMS bias correction (0.037%).

## Data availability

All relevant data are included in the paper and in the Supporting Information. The data-sets created as part of this study or used for data analysis are available upon reasonable request from the corresponding author.

### Supplementary Information

Supplementary Information.
