# Supplementary material for: The 146Sm half-life re-measured: consolidating the chronometer for events in the early Solar System
Source: Sci Rep. 2024 Aug 1;14:17436. doi: 10.1038/s41598-024-64104-6 (PMC11294585; doi:10.1038/s41598-024-64104-6)
Supplement: Supplementary file 1 — Supplementary Information. [file 41598_2024_64104_MOESM1_ESM.pdf]

Supplementary Information

The ^{146}Sm half-life re-measured: Consolidating the chronometer for events in the early Solar System.

Nadine M. Chiera, Peter Sprung, Yuri Amelin, Rugard Dressler, Dorothea Schumann, Zeynep Talip

SI 1 Protocols for gravimetrically determined aliquots

SI 1.1 Total amount of retrieved Sm master-solution

Table SI 1. The total amount of 12.013 423(23) g Sm master-solution retrieved from the irradiated tantalum samples was calculated as the difference between gross weight of the vial with Sm master-solution (+ Sm solution) and the tare of the vial (Vial). The means and combined standard uncertainty of twelve consecutive weightings are indicated.

Weighting #	Vial (g)	+ Sm solution (g)
1	6.732 87	18.746 26
2	6.732 83	18.746 29
3	6.732 86	18.746 32
4	6.732 93	18.746 31
5	6.732 95	18.746 32
6	6.733 06	18.746 33
7	6.732 85	18.746 35
8	6.732 88	18.746 36
9	6.732 90	18.746 36
10	6.732 94	18.746 35
11	6.732 91	18.746 37
12	6.732 94	18.746 38
Mean	6.732 910	18.746 333
Uncert.	0.000 020	0.000 013

SI 1.2 Dilution of samarium standard for MC-ICP-MS

Table SI 2. Gravimetrically determined concentration of the diluted samarium standard solution made for MC-ICP-MS. The mass of the used vial (Vial) , after adding 0.28 M HNO_3 (+ HNO_3), and after adding the Sm LGC-standard (+ Sm standard) are given. The means and combined standard uncertainty of twelve consecutive weightings are indicated. The total mass of the produced solution was calculated to be 49.911 076(35) g containing 0.159 327(33) g of the Sm LGC-standard. The samarium concentration of this solution is 21.209(43) nmol/g.

Weighting #	Vial (g)	+ HNO_3 (g)	+ Sm standard (g)
1	13.538 40	63.290 32	63.449 55
2	13.538 48	63.290 30	63.449 55
3	13.538 61	63.290 22	63.449 60
4	13.538 69	63.290 22	63.449 65
5	13.538 62	63.290 25	63.449 61
6	13.538 41	63.290 20	63.449 59
7	13.538 58	63.290 49	63.449 73
8	13.538 57	63.290 39	63.449 75
9	13.538 61	63.290 31	63.449 66
10	13.538 62	63.290 26	63.449 68
11	13.538 50	63.290 37	63.449 62
12	13.538 59	63.290 34	63.449 60
Mean	13.538 557	63.290 306	63.449 633
Uncert.	0.000 028	0.000 026	0.000 020

SI 1.3 Dilution of gadolinium standard for MC-ICP-MS

Table SI 3. Gravimetrically determined concentration of the diluted gadolinium standard solution made for MC-ICP-MS. The mass of the used mass (Vial) , after adding 0.28 M HNO_3 (+ HNO_3), and after adding the Gd Specpure-standard (+ Gd standard) are given. The means and combined standard uncertainty of three consecutive weightings are indicated. A reference gadolinium standard solution Specpure by Thermo Scientific Chemicals (1000 µg/mL natural gadolinium in 5 % HNO_3, LOT: 223965) with a certified content of 0.990 00(150) mg/mL was used. The total mass of the produced solution was calculated to be 9.992 190(26) g containing 6.014 967(60) g of the gadolinium standard. The gadolinium concentration of this solution is 3.7898(58) µmol/g.

Weighting #	Vial (g)	+ HNO_3 (g)	+ Gd standard (g)
1	6.865 68	10.842 81	16.857 84
2	6.865 67	10.842 91	16.857 88
3	6.865 65	10.842 95	16.857 85
Mean	6.865 667	10.842 890	16.857 857
Uncert.	0.000 017	0.000 056	0.000 020

SI 1.4 Preparation of samarium samples for MC-ICP-MS

Table SI 4. Composition of the sample solutions used by the reverse isotope dilution and gravimetric standard addition with an additional internal standard ICP-MS techniques. To each Sm master-solution aliquot ("Sm master"), aliquots of the diluted samarium standard ("Sm dil. std.") solution and the diluted gadolinium standard ("Gd dil. std.") solution were added in vials containing the given amounts of 0.28 M HNO_3. The means with its standard uncertainties of twelve consecutive weightings are reported.

Addition	Sample 0 (g)	Sample 1 (g)	Sample 2 (g)	Sample 3 (g)	Sample 4 (g)
Sm master	0.100 707(11)	0.100 608(11)	0.101 488(15)	0.101 942(16)	0.101 648(11)
Sm dil. std.	0	0.302 539(11)	4.933 390(18)	9.837 078(13)	14.809 543(20)
Gd dil. std.	0.101 178(12)	0.099 311(11)	0.100 891(17)	0.101 868(14)	0.101 629(21)
HNO_3	14.703 107(14)	14.576 093(17)	9.942 620(21)	5.014 104(13)	0

SI 1.5 Gravimetric determination of the TE-TIMS IDMS sample

Table SI 5. Gravimetrically determined concentration of the diluted samarium solutions ("CalMix") used for TE-TIMS IDMS. The averaged mass together with their uncertainties of the initial vial (Vial), after adding an aliquot of the Sm Ames-solution, and after adding the Sm TIMS-solution are given. The total content with its uncertainties of the Sm Ames-solution(Ames content) as well as of the Sm TIMS-solution (^{146}Sm content) was calculated as the difference between the given gross weights.

CalMix #	Vial (g)	+ Ames aliquot (g)	+ ^{146}Sm (g)	Ames content (g)	^{146}Sm content (g)
10	11.823 570(17)	12.332 680(17)	12.383 320(16)	0.509 110(23)	0.050 640(23)
13	11.418 930(17)	11.927 520(17)	11.998 860(16)	0.508 590(23)	0.071 340(23)
14	11.504 180(17)	12.013 180(17)	12.114 700(16)	0.509 000(23)	0.101 520(23)
15	11.441 520(17)	11.949 420(17)	12.153 320(16)	0.507 900(23)	0.203 900(23)
16	11.576 820(17)	12.085 670(17)	12.594 450(16)	0.508 850(23)	0.508 780(23)

SI 1.6 Gravimetric determination of the Sm TIMS-sample after TIMS

Table SI 6. Gravimetrically determined amount of the remaining Sm TIMS-sample returned from ANU to PSI. The amount of 6.999 753(23) g of the returned Sm TIMS-solution was calculated as the difference between gross weight (Sm-TIMS + vial) and emptied vial (Vial). The means and combined standard uncertainty of six consecutive weightings are indicated.

Weighting #	Sm-TIMS + vial (g)	Vial (g)
1	38.297 86	31.298 06
2	38.297 82	31.298 04
3	38.297 81	31.298 06
4	38.297 81	31.298 02
5	38.297 73	31.298 07
6	38.297 78	31.298 04
Mean	38.297 802	31.298 048 3
Uncert.	0.000 020	0.000 009 7

SI 1.7 Sm master-solution aliquot for molecular plating

Table SI 7. The total amount of 6.293 167(23) g Sm master-solution used for molecular plating was calculated as the difference between gross weight (+ Sm aliquot) and tare (Vial). The means and combined standard uncertainty of twelve consecutive weightings are indicated.

**Weighting** #	**Vial** (g)	**+ Sm aliquot** (g)
1	6.732 87	13.026 12
2	6.732 83	13.026 12
3	6.732 86	13.026 06
4	6.732 93	13.026 08
5	6.732 95	13.026 07
6	6.733 06	13.026 02
7	6.732 85	13.026 02
8	6.732 88	13.026 06
9	6.732 90	13.026 07
10	6.732 94	13.026 09
11	6.732 91	13.026 09
12	6.732 94	13.026 12
Mean	6.732 910	13.026 077
Uncert.	0.000 020	0.000 012

SI 2 Molecular plating

SI 2.1 Holder for ^{145}Sm γ-measurements before and after molecular plating

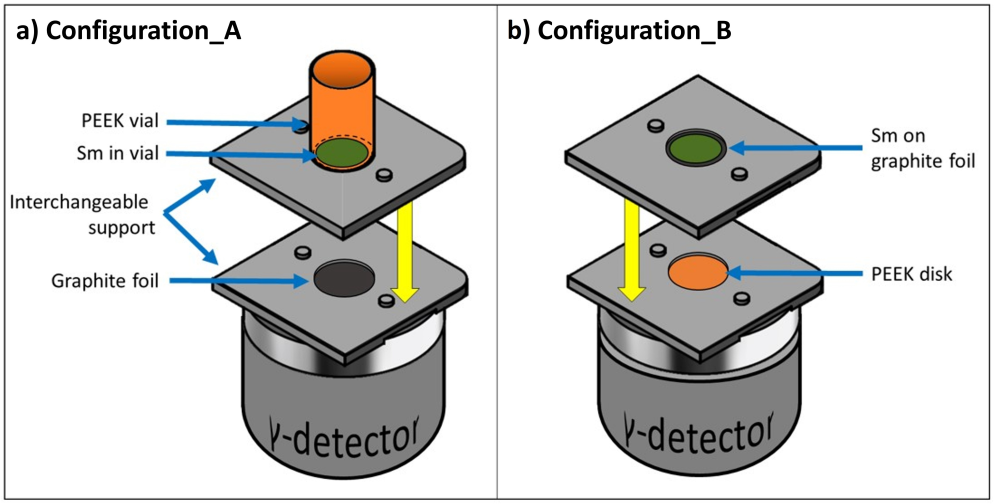

Figure SI 1. Schematic drawing of the custom-made holder made of two interchangeable parts, namely a) Configuration_A and b) Configuration_B, used to perform γ-measurement_A and γ-measurement_C, and γ-measurement_B, respectively.

SI 2.2 Molecular plating efficiency

The γ-spectra of ^{145}Sm before molecular plating (γ-measurement_A, in Configuration_A of Figure SI 1), and ^{145}Sm deposited on the graphite foil (γ-measurement_B, in Configuration_B of Figure SI 1) are shown in Figure SI 2. For sake of completeness, the background spectrum is shown as well. Details on the deduced count rates are given in Table SI 8.

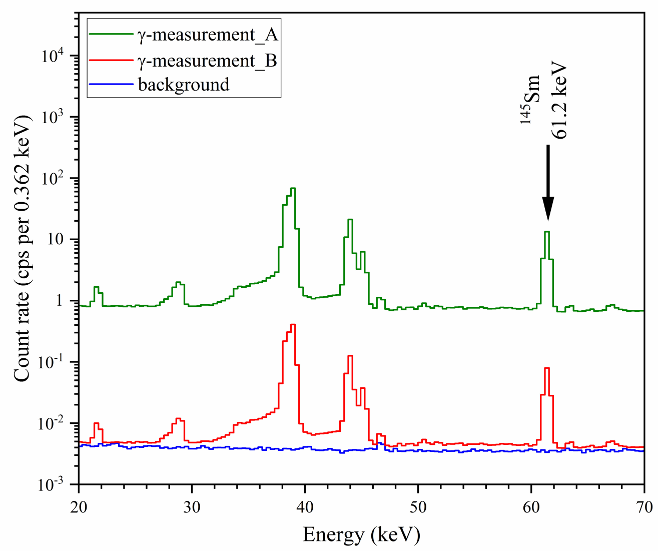

Figure SI 2. Black line: γ-spectrum of ^{145}Sm before molecular plating (γ-measurement_A); red line: γ-spectrum of ^{145}Sm deposited on the graphite foil (γ-measurement_B); blue line: background. The energy bin is 0.362 keV. The reference peak used to calculate the efficiency of the molecular plating is indicated.

Table SI 8. Count rate (in cps) at 61.2 keV corresponding to the γ-ray measurements of ^{145}Sm contained in the ^{146}Sm aliquot before molecular plating (γ-measurement_A) and in the deposited samarium layer (γ-measurement_B). The exact dates (in dd.mm.yyyy format) at which the measurements were performed are given. The real time (t_{real}, in second) and live-time (t_{live}, in second) of each measurement are reported as well.

	**Date** (dd.mm.yyyy)	t_{real} (s)	t_{live} (s)	**Count rate** (cps)
γ-measurement_A	24.06.2021	3600	3585	8.525(50)
γ-measurement_B	11.08.2021	601200	601015	0.124 62(54)

SI 2.3 γ-Spectrometric measurement of Sm TIMS-solution after TIMS

The γ-spectrum of ^{145}Sm contained in the Sm TIMS-solution (γ-measurement_C, in Configuration_A of Figure SI 1) is shown in Figure SI 3. For comparison, the γ-spectrum of ^{145}Sm contained in an aliquot of Sm master-solution (γ-measurement_A, in Configuration_A of Figure SI 3) is shown as well. Details on the deduced count rates are given in Table SI 9.

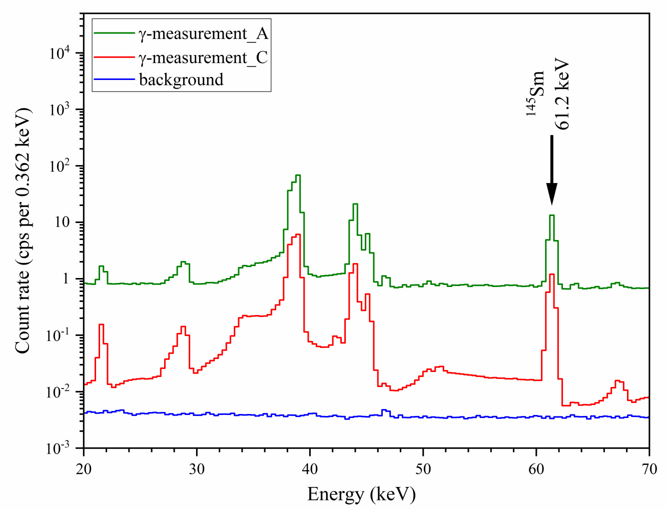

Figure SI 3. Black line: γ-spectrum of ^{145}Sm contained in an aliquot of 6.293 17(2) g of Sm master-solution (γ-measurement_A, @ 24 June 2021); orange line: γ-spectrum of ^{145}Sm contained in 6.999 75(5) g of Sm TIMS-solution (γ-measurementA_C, @ 03 March 2022); blue line: background. The energy bin is 0.362 keV. The ^{145}Sm reference peak is indicated.

Table SI 9. Count rate (in cps) at 61.2 keV corresponding to γ-measurement_A (Sm master-solution aliquot, 6.293 17(2) g) and γ-measurement_C (Sm TIMS-solution, 6.999 75(5) g). The exact dates (in dd.mm.yyyy format) at which the measurements were performed are given. The real time (t_{real}, in second) and live-time (t_{live}, in second) of each measurement are reported as well.

	**Date** (dd.mm.yyyy)	t_{real} (s)	t_{live} (s)	**Count rate** (cps)
γ-measurement_A	24.06.2021	3600	3585	8.525(50)
γ-measurement_C	03.03.2022	903673	431256	2.0954(22)

SI 3 α-Spectrometric measurements

α-spectrometric measurements were performed by counting at a defined solid angle. Figure SI 4 shows the count rate of the used ^{241}Am PTB reference source recorded with the very same PIPS detector used for the ^{146}Sm α-measurement (A-450-21AM, Canberra; Detector sensitive area 450 mm^2, sample surface to detector distance = 10.4 mm, nominal FWHM = 21 keV, counting time = 500 s).

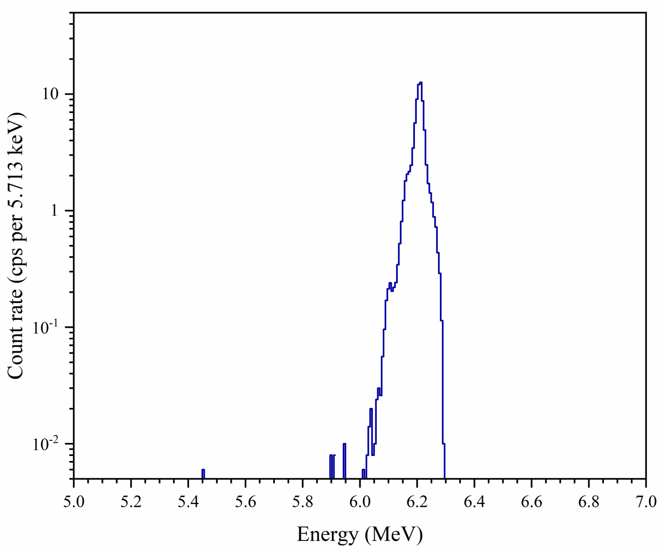

Figure SI 4. α-spectrum of the ^{241}Am PTB reference source used for the efficiency calibration. The energy bin corresponds to 5.713 keV.

The α-peaks at 2.87 MeV and 3.18 MeV have a FWHM of 27.65 keV and 31.87 keV, respectively. These values, calculated by the Genie 2000 Alpha Analysis Software, are close to the 21 keV nominal resolution of the used PIPS detector specified by the producer. This indicates a thin deposition layer of the ^{146}Sm sample. Peak-fits of the recorded α-spectrum were performed with the Origin 2021b NLFit tool (OriginLab Corporation) by applying the following system of equations (SI 2)–(SI 7), as suggested in Pommé and Caro Marroyo [1] to parameterize the α-peak-shape y_{Peak} for each of the ^{146}Sm, ^{147}Sm, and ^{148}Gd peaks:

$$z_1 = \frac{1}{2} \times \left(\frac{w}{t_1}\right)^2 + \left(\frac{x-x_c}{t_1}\right) \tag{SI 1}$$

$$z_2 = \frac{1}{2} \times \left(\frac{w}{t_2}\right)^2 + \left(\frac{x-x_c}{t_2}\right) \tag{SI 2}$$

$$z_3 = \frac{1}{2} \times \left(\frac{w}{t_3}\right)^2 + \left(\frac{x-x_c}{t_3}\right) \tag{SI 3}$$

$$y_1 = \frac{\text{erfc}(z_1) \times A \times K}{2 \times t_1 \times (1+K+L)} \times \mathbf{e}^{z_1} \tag{SI 4}$$

$$y_2 = \frac{\text{erfc}(z_2) \times A}{2 \times t_2 \times (1+K+L)} \times \mathbf{e}^{z_2} \tag{SI 5}$$

$$y_3 = \frac{\text{erfc}(z_3) \times A \times L}{2 \times t_3 \times (1+K+L)} \times \mathbf{e}^{z_3} \tag{SI 6}$$

$$y_{\text{Peak}} = y_1 + y_2 + y_3 \tag{SI 7}$$

Here, $x_{c-\text{Peak}}$ denotes the peak center, A_{Peak} the fitted count rate area, w the Gaussian width component, and t_1, t_2, and t_3 the three independent low-energy tailing parameters, with K_{Peak} and L_{Peak} the relative intensities of the first and third tailing with respect to the second one. The function erfc(z), defined in the Origin 2021b NLFit software as

$$\text{erfc}(z) = \frac{1}{\sqrt{\pi}} \int_z^{\infty} \mathbf{e}^{-\frac{t^2}{2}}\, dt \tag{SI 8}$$

denote the Gauss complementary error functions. The entire fitted spectrum y is composed by 1) the sum of the three y_{Peak} peak areas of the ^{146}Sm, ^{147}Sm, and ^{148}Gd α-activity, i.e., $y_{P(Sm-146)}$, $y_{P(Sm-147)}$, and $y_{P(Gd-148)}$, respectively; 2) the area contribution y_E of the low-energetic electronic noise having an exponential factor η and an amplitude a_E; and 3) a constant background y_0. The mathematical expression for y is given in the following system of equations (SI 9) and (SI 9):

$$y_E = \frac{a_E}{\eta} \times \mathbf{e}^{-\frac{x}{\eta}} y = y_0 + y_E + y_{P(Sm-146)} + y_{P(Sm-147)} + y_{P(Gd-148)} \tag{SI 9}$$

The fitted parameters, i.e., the optimized parameters that allow for calculating at each energy bin the corresponding signal height y that best reproduce the experimental α-spectrum, are indicated in Table SI 10.

Table SI 10. Parameters obtained with the Origin 2021b NLFit tool for the fit of the α-spectrum plotted in Figure 2 (only uncertainties from the fitting procedure are given). In the last line the Coefficient of determination (R-squared) for the fit is also displayed.

**Parameter**	**^{146}Sm fit-peak**	**^{147}Sm fit-peak**	**^{148}Gd fit-peak**
	individual peak shape parameter		
A_{Peak}	0.01263(16) cps × keV	0.00012(2) cps × keV	0.01345(20) cps × keV
$x_{c-\text{Peak}}$	2471.29(15) keV	2256.46(290) keV	3196.80(9) keV
K_{Peak}	2.85(33)	2.85(33)	0.68(8)
L_{Peak}	0.37(6)	0.37(6)	0.83(4)
	shared peak-fit parameters		
w	7.61(19) keV		
t_1	10.01(43) keV		
t_2	38.34(228) keV		
t_3	481.81(4064) keV		
	noise and background parameters		
y_0	$3.16(37) \cdot 10^{-7}$ cps		
η	168.0(80) keV		
a_E	1.42(42) cps × keV		
R^2 (CoD)	0.98018		

By taking into account the energy bin width of 5.713 keV, it is possible to obtain for each peak the total count rate C_{Peak}. Final results of the count rate in the three ^{146}Sm, ^{147}Sm, and ^{148}Gd α-peaks are reported in Table SI 11.

Table SI 11. Count rate area (A_{Peak}) of the ^{146}Sm, ^{147}Sm, and ^{148}Gd α-peaks for the histogram plotting the count rate per energy bin (in eV). The total count rate (C_{Peak}) of the ^{146}Sm, ^{147}Sm, and ^{148}Gd α-peaks for a histogram plotting the count rate per channel is reported. The uncertainties on the parameter A_{Peak} are derived from the fitting procedure. For the parameter C_{Peak}, the combined uncertainties of the fit and the Poisson counting statistics are given.

	**A_{Peak}** (cps × eV)	**C_{Peak}** (cps)
^{146}Sm	12.63(16)	$2.210(36) \cdot 10^{-3}$
^{147}Sm	0.125(24)	$0.0219(47) \cdot 10^{-3}$
^{148}Gd	13.45(20)	$2.354(41) \cdot 10^{-3}$

It follows that the count rate for the decay of ^{146}Sm is 0.002210(36) cps. By correlating the count rate of the ^{146}Sm peak to the count rate associated to the peak of the calibrated ^{241}Am reference standard source (PTB, calibration reference No PTB-6.11-2016-1769), an activity of 15.00(34) mBq for ^{146}Sm was obtained (see Table SI 12).

Table SI 12. Activity (in Bq) of the ^{146}Sm deposited on the graphite foil. The activity of the ^{241}Am reference standard source used for efficiency calibration is indicated as well. For each measurement, the real time (t_{real}, in second) and live-time (t_{live}, in second) is given. For sake of completeness, the activities of ^{147}Sm and ^{148}Gd are reported as well. The energy range considered for the calculation of the count rate (in cps) of each α-peak is specified.

	t_{real} (s)	t_{live} (s)	Energy range (MeV)	Count rate (cps)	Activity (Bq)
^{241}Am	500	501	5.3 to 5.6	78.84(56)	534.9(55)[a]
^{146}Sm	$5{\cdot}10^{6}$	$5{\cdot}10^{6}$	1.0 to 3.2	$2.210(36){\cdot}10^{-3}$	$1.500(34){\cdot}10^{-2}$
^{147}Sm	$5{\cdot}10^{6}$	$5{\cdot}10^{6}$	1.0 to 3.2	$2.19(47){\cdot}10^{-5}$	$1.48(35){\cdot}10^{-4}$
^{148}Gd	$5{\cdot}10^{6}$	$5{\cdot}10^{6}$	1.0 to 3.2	$2.354(41){\cdot}10^{-3}$	$1.597(37){\cdot}10^{-2}$

[a] Activity of the reference standard source at the date of the efficiency calibration (20 July 2021), calculated using 432.6(6) a as the half-life of ^{241}Am Nesaraja [2].

SI 4 Mass-spectrometric measurements

SI 4.1 MC-ICP-MS results

Table SI 13. Isotope ratios determined by MC-ICP-MS. All values are corrected for mass fractionation and signal baseline. The baseline correction was performed using an exponential-law mimic well the washout behavior of the measured blank values of ^{148}Sm/^{147}Sm before and after the measurement of an analyte solution using wash solutions. The isobaric interference for the masses 146, 148, and 150 (in the table marked with $^{♀}$) were corrected on the basis of the ^{145}Nd signal assuming a natural neodymium isotopy. The isotope ratios of ^{143}Nd/^{146}Sm and ^{145}Nd/^{146}Sm are derived without any interference correction from reduced data sets in which negative values after baseline correction were excluded (results below the quantification limit are placed in curly brackets). The uncertainties refers to standard deviations of a set of 9 repeated individual analyses per sample each consisting of sixty 7.5-s-long signal integration. Note that any inter- and intra-day variability of the empirical relation between the samarium and gadolinium mass fractionation magnitude is intrinsically incorporated in the presented results given that three distinct analytical sessions are included in the average results.

Ratio	Sample 0	Sample 1	Sample 2	Sample 3	Sample 4
^{147}Sm/^{146}Sm$^{♀}$	9.061(11)	18.692(39)	165.061(91)	319.436(72)	476.78(25)
^{148}Sm$^{♀}$/^{146}Sm$^{♀}$	1.9084(60)	9.130(30)	118.883(81)	234.674(59)	352.69(22)
^{149}Sm/^{146}Sm$^{♀}$	0.9534(59)	9.831(37)	144.76(12)	287.16(12)	432.28(33)
^{150}Sm$^{♀}$/^{146}Sm$^{♀}$	3.7480(83)	8.488(17)	80.550(82)	156.627(98)	234.16(21)
^{147}Sm/^{155}Gd	1.966(30)	4.108(64)	36.17(58)	70.48(98)	105.7(19)
^{148}Sm$^{♀}$/^{155}Gd	0.4142(71)	2.006(29)	26.05(43)	51.78(73)	78.2(14)
^{149}Sm/^{155}Gd	0.2069(42)	2.160(31)	31.72(52)	63.36(91)	95.9(17)
^{150}Sm$^{♀}$/^{155}Gd	0.813(13)	1.865(29)	17.65(30)	34.56(50)	51.93(95)
^{147}Sm/^{157}Gd	1.861(28)	3.887(59)	34.23(55)	66.70(92)	100.1(18)
^{148}Sm$^{♀}$/^{157}Gd	0.3920(66)	1.899(27)	24.65(40)	49.00(69)	74.0(13)
^{149}Sm/^{157}Gd	0.1958(39)	2.044(29)	30.02(49)	59.96(85)	90.8(16)
^{150}Sm$^{♀}$/^{157}Gd	0.770(12)	1.765(28)	16.70(28)	32.71(47)	49.16(89)
^{147}Sm/^{158}Gd	1.173(17)	2.450(37)	21.58(34)	42.04(57)	63.1(11)
^{148}Sm$^{♀}$/^{158}Gd	0.2470(41)	1.197(17)	15.54(25)	30.88(42)	46.66(83)
^{149}Sm/^{158}Gd	0.1234(25)	1.289(18)	18.92(30)	37.79(53)	57.2(10)
^{150}Sm$^{♀}$/^{158}Gd	0.4852(77)	1.113(17)	10.53(17)	20.61(29)	30.98(56)
^{143}Nd/^{146}Sm	$4.4(16){\cdot}10^{-4}$	$3.2(18){\cdot}10^{-4}$	$\{8.8(83){\cdot}10^{-5}\}$	$\{8.5(58){\cdot}10^{-5}\}$	$\{1.33(92){\cdot}10^{-5}\}$
^{145}Nd/^{146}Sm	$4.0(11){\cdot}10^{-4}$	$2.0(17){\cdot}10^{-4}$	$\{1.9(27){\cdot}10^{-4}\}$	$3.1(13){\cdot}10^{-4}$	$\{3.3(23){\cdot}10^{-4}\}$

SI 4.2 TIMS results

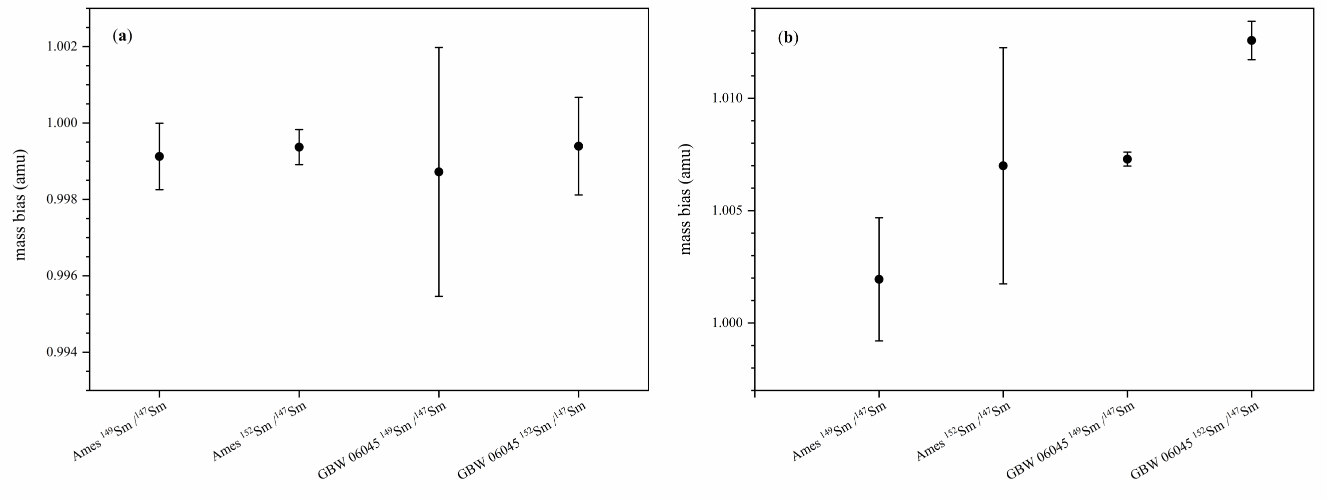

Figure SI 5. Determination of mass bias in samarium isotope analyses by TIMS using a) the total evaporation method (TE-TIMS, left panel) and b) the Langmuir corrected incipient emission (IE-TIMS, right panel) method, following the principles of the methods described in Chang et al. [3]. Two samarium reference materials (Sm Ames-solution and Sm GBW-solution) were loaded into a double filament (Re-Re) assembly. Plotted is the mass bias coefficient of a power-law mass bias correction deduced from the given samarium isotopes. Each point represents an average together with the $k = 2$ extended standard uncertainty of 5 measurements from separate filament loads for each reference material. These measurements yielded precise data using TE-TIMS with systematic uncertainty of less than 0.1 %, while the IE-TIMS data are not reproducible within their uncertainty limits. The obtained average mass bias is 0.999 15(52) using an arithmetic mean or 0.999 32(39) using a weighted mean. (The results for the IE-TIMS are 1.0072(73) arithmetic mean and 1.007 84(280) weighted mean, respectively). The result of the weighted mean of the TE-TIMS data was therefore chosen as the mass-independent coefficient for the mass bias correction in this study.

Table SI 14. Isotope ratios determined by TE-TIMS. Values are corrected for mass bias as described in the main text. The values and uncertainties (by $k = 2$ extended standard uncertainty) of the CalMix solutions (CalMix #) based on a single measurement of a filament load and represent time integrated signals of the Faraday-cup readouts. The value and uncertainty (by $k = 2$ extended standard uncertainty) of the pure Sm TIMS-solution (Sm TIMS) refers to a set of 5 measurements from separate filament loads.

**Sample**	**^{146}Sm/^{147}Sm**	**^{149}Sm/^{147}Sm**	**^{152}Sm/^{147}Sm**	**^{143}Nd/^{146}Sm**	**^{145}Nd/^{146}Sm**
Calmix 10	0.008 540(11)	0.857 67(67)	1.6463(32)	$-8.3(126)\cdot10^{-4}$	$-5.0(13)\cdot10^{-3}$
Calmix 13	0.011 669 2(66)	0.833 06(65)	1.5903(31)	$-1.00(47)\cdot10^{-3}$	$-3.2(416)\cdot10^{-5}$
Calmix 14	0.015 831 7(73)	0.802 24(63)	1.5248(30)	$7.5(26)\cdot10^{-4}$	$1.24(23)\cdot10^{-3}$
Calmix 15	0.027 870(11)	0.713 42(56)	1.3369(26)	$1.05(11)\cdot10^{-3}$	$-2.6(11)\cdot10^{-4}$
Calmix 16	0.050 413(20)	0.547 11(43)	0.9851(19)	$1.34(35)\cdot10^{-4}$	$1.10(35)\cdot10^{-4}$
Sm TIMS	0.110 35(33)	0.104 35(20)	0.048 16(23)	$5.9(91)\cdot10^{-5}$	$4.2(63)\cdot10^{-5}$

SI 5 Uncertainty calculation for averages

For a realistic estimate of the uncertainty of calculated mean values, two independent components must be taken into account: 1) the intrinsic uncertainty, which represents the variation of the measurement data, and 2) the extrinsic uncertainty, which results from the uncertainties of the individual measurements. At this point, special care must be taken to ensure that these uncertainties do not include uncertainty components of commonly used quantities such as the concentration of standard solutions or the half-life of reference isotopes. The uncertainty contribution of such quantities would be reduced by the averaging procedure, which would lead to unrealistically low uncertainties. The uncertainties of such components must be considered separately after averaging. In the following, a set of N data points x_i with associated uncertainties u_i is used.

SI 5.1 Arithmetic mean

The combined standard uncertainty υ_A of the arithmetic mean μ_A is obtained from the extrinsic υ_{ext} and the intrinsic υ_{int} uncertainties, respectively, and calculated as follow

$$\begin{aligned}
\mu_A &= \frac{\sum_{i=1}^{N} x_i}{N} \\
\upsilon_{ext}^2 &= \frac{\sum_{i=1}^{N} u_i^2}{N^2} \\
\upsilon_{int}^2 &= \frac{t_{\alpha,N-1}^2}{N-1} \times \frac{\sum_{i=1}^{N} (x_i-\mu)^2}{N} \\
\upsilon^2 &= \upsilon_{ext}^2 + \upsilon_{int}^2
\end{aligned}$$

with $t_{\alpha/2;N-1}$ the Student's t-distribution to a confidence limit $1-\alpha$ and $N-1$ degrees of freedom. The confidence limit $1-\alpha$ was chosen in a way to create a symmetrical coverage interval of a standard normal distribution with a width of 2.

SI 5.2 Weighted mean

In this case, the individual uncertainties u_i are used to calculate the corresponding weights $\omega_i = 1/u_i^2$ for each data point x_i. The approach to estimate the variance of the weighted mean proposed by Cochran [4], Endlich et al. [5], and Gatz and Smith [6] is used to calculate the intrinsic uncertainties υ_{int} (see Nadderd et al. [7] for a summary of the respective references). Similar to the arithmetic mean, the combined standard uncertainty υ_w of the weighted mean μ_w results from the extrinsic υ_{ext} and the intrinsic υ_{int} uncertainties, respectively, and are calculated as follow

$$\begin{aligned}
\mu_w &= \frac{\sum_{i=1}^{N} \omega_i x_i}{\sum_{i=1}^{N} \omega_i} \\
\upsilon_{ext}^2 &= \frac{\sum_{i=1}^{N} \omega_i^2 u_i^2}{\left(\sum_{i=1}^{N} \omega_i\right)^2} = \frac{1}{\sum_{i=1}^{N} \omega_i} \\
\upsilon_{int}^2 &= \frac{N \times t_{\alpha/2;N-1}^2}{N-1} \times \frac{\sum_{i=1}^{N} \omega_i^2 (x_i-\mu_w)^2}{\left(\sum_{i=1}^{N} \omega_i\right)^2} \\
\upsilon^2 &= \upsilon_{ext}^2 + \upsilon_{int}^2
\end{aligned}$$

with $t_{\alpha/2;N-1}$ the Student's t-distribution to a confidence limit $1-\alpha$ and $N-1$ degrees of freedom. The confidence limit $1-\alpha$ was chosen in a way to create a symmetrical coverage interval of a standard normal distribution with a width of 2.

Under ideal conditions, both the intrinsic and extrinsic uncertainty components represent the same variation in the measured value. Therefore, the methods used here are conservative estimates of the combined standard uncertainty. It does not overestimate the uncertainty by more than 41 % if the above-mentioned ideal case occurs. Normally, however, the intrinsic and extrinsic uncertainties differ by factors or even orders of magnitude. Under these circumstances, this method guarantees the most realistic estimate of the uncertainty.

References

1. S. Pommé and B. Caro Marroyo. Improved peak shape fitting in alpha spectra. *Applied Radiation and Isotopes*, 96:148–153, February 2015. ISSN 09698043. doi: 10.1016/j.apradiso.2014.11.023. URL https://linkinghub.elsevier.com/retrieve/pii/S0969804314004175.

2. C. D. Nesaraja. Nuclear Data Sheets for A = 241. *Nuclear Data Sheets*, 130:183–252, December 2015. ISSN 00903752. doi: 10.1016/j.nds.2015.11.004. URL https://linkinghub.elsevier.com/retrieve/pii/S0090375215000587.

3. Tsing-Lien Chang, Mo-Tian Zhao, Wen-Jun Li, Jun Wang, and Qiu-Yu Qian. Absolute isotopic composition and atomic weight of samarium. *International Journal of Mass Spectrometry*, 218(2):167–172, July 2002. ISSN 13873806. doi: 10.1016/S1387-3806(02)00665-6. URL https://linkinghub.elsevier.com/retrieve/pii/S1387380602006656.

4. Wiliam G. Cochran. *Sampling Techniques*. Wiley Series in Probability and Statistics. John Wiley & Sons Inc, 3rd edition, 1977. ISBN 978-0-471-16240-7.

5. R. M. Endlich, B. P. Eymon, R. J. Ferek, A. D. Valdes, and C. Maxwell. Statistical Analysis of Precipitation Chemistry Measurements over the Eastern United States. Part I: Seasonal and Regional Patterns and Correlations. *Journal of Applied Meteorology*, 27(12):1322–1333, December 1988. ISSN 0894-8763, 1520-0450. doi: 10.1175/1520-0450(1988)027<1322:SAOPCM>2.0.CO;2. URL http://journals.ametsoc.org/doi/10.1175/1520-0450(1988)027<1322:SAOPCM>2.0.CO;2.

6. Donald F. Gatz and Luther Smith. The standard error of a weighted mean concentration—I. Bootstrapping vs other methods. *Atmospheric Environment*, 29(11):1185–1193, June 1995. ISSN 13522310. doi: 10.1016/1352-2310(94)00210-C. URL https://linkinghub.elsevier.com/retrieve/pii/135223109400210C.

7. L. Nadderd, J. Milosevic, and F. Wang. Statistical uncertainties of the $v_n 2k$ harmonics from Q cumulants. *Physical Review C*, 104(3):034906, September 2021. ISSN 2469-9985, 2469-9993. doi: 10.1103/PhysRevC.104.034906. URL https://link.aps.org/doi/10.1103/PhysRevC.104.034906.
